# Health Services Use and End‐of‐Life Outcomes Among People with Dementia and Multiple Chronic Conditions

**DOI:** 10.1002/alz.090921

**Published:** 2025-01-09

**Authors:** Hyosin Kim, Haiqun Lin, Anum Zafar, Maria J. Lopez, Weiyi Xia, Olga F Jarrín Montaner

**Affiliations:** ^1^ Oregon State University, Corvallis, OR USA; ^2^ Rutgers, The State University of New Jersey, Newark, NJ USA; ^3^ Rutgers, The State University of New Jersey, New Brunswick, NJ USA; ^4^ Rutgers, The State University of New Jersey, Piscataway, NJ USA

## Abstract

**Background:**

Individuals with dementia often have one or more chronic conditions, and the disease burden and care experience may differ based on the plurality of chronic conditions. We aim to describe the individual characteristics, health care use, and place of death for individuals with dementia and multiple comorbidities.

**Method:**

A retrospective cohort of individuals who died in 2019 with dementia, and were continuously enrolled in Medicare for at least three years. We constructed a database summarizing health services utilization during the last three years of life and end‐of‐life outcomes by harmonizing inpatient and hospice claims, and assessment data from post‐acute and long‐term care settings. Individuals were categorized by number of comorbidities. Bivariate association analyses were performed to assess the statistical differences in the variables of interest by comorbidity levels.

**Result:**

Beneficiaries with dementia (n = 933,618) had a median of seven additional comorbidities [IQR 5‐8]. Fifteen percent had three or fewer comorbidities. Those with four or more comorbidities were significantly more likely to be hospitalized (85% vs. 67%), admitted to a nursing home (66% vs. 51%), and use home health care (56% vs. 49%) during the last three years of life compared to those with fewer comorbidities. Inpatient death without hospice (29% vs. 18%) was higher among those with four or more comorbidities compared to those with fewer comorbidities. A greater use of hospice was observed within beneficiaries with three or less comorbidities in addition to dementia (71% vs. 62%).

**Conclusion:**

Individuals with dementia and more than four additional chronic conditions used more health care services in both inpatient and home care settings, while they underutilized hospice services at the time of death compared to those with fewer chronic conditions. Policies aimed at enhancing access to home‐ and community‐based services for individuals with dementia and multiple comorbidities have the potential to enhance end‐of‐life care experiences and contribute to care quality for people living with dementia and support for their family caregivers.

